# Changes in Intestinal Microbiota and Predicted Metabolic Pathways During Colonic Fermentation of Mango (*Mangifera indica* L.)—Based Bar Indigestible Fraction

**DOI:** 10.3390/nu12030683

**Published:** 2020-03-03

**Authors:** Wilbert Gutiérrez-Sarmiento, Sonia Guadalupe Sáyago-Ayerdi, Isabel Goñi, Federico Antonio Gutiérrez-Miceli, Miguel Abud-Archila, José del Carmen Rejón-Orantes, Reiner Rincón-Rosales, Betsy Anaid Peña-Ocaña, Víctor Manuel Ruíz-Valdiviezo

**Affiliations:** 1 Tecnológico Nacional de México/IT de Tuxtla Gutiérrez, Carretera Panamericana km. 1080, Tuxtla Gutiérrez CP 29050, Chiapas, Mexico; 2 Tecnológico Nacional de México/IT de Tepic, Av. Tecnológico 2595, Nayarit, Tepic CP 63175, Mexico; 3 Department Nutrition and Food Science, Faculty of Pharmacy, University Complutense of Madrid, 28040 Madrid, Spain; 4 Pharmacobiology Experimental Laboratory, Faculty of Human Medicine, Universidad Autónoma de Chiapas, Calle Central-Sur S/N, San Francisco, Tuxtla Gutiérrez 29090, Chiapas, Mexico

**Keywords:** “Ataulfo” mango, in vitro colonic fermentation, intestinal microbiota, 16S rRNA gene, bioinformatics

## Abstract

Mango (*Mangifera indica* L.) peel and pulp are a source of dietary fiber (DF) and phenolic compounds (PCs) that constituent part of the indigestible fraction (IF). This fraction reaches the colon and acts as a carbon and energy source for intestinal microbiota. The effect of mango IF on intestinal microbiota during colonic fermentation is unknown. In this study, the isolated IF of a novel ‘Ataulfo’ mango-based bar (snack) UV-C irradiated and non-irradiated (UVMangoB and MangoB) were fermented. Colonic fermentation occurred in vitro under chemical-enzymatic, semi-anaerobic, batch culture and controlled pH colonic conditions. Changes in the structure of fecal microbiota were analyzed by 16s rRNA gene Illumina MiSeq sequencing. The community´s functional capabilities were determined in silico. The MangoB and UVMangoB increased the presence of *Faecalibacterium*, *Roseburia*, *Eubacterium*, *Fusicatenibacter*, *Holdemanella*, *Catenibacterium*, *Phascolarctobacterium*, *Buttiauxella*, *Bifidobacterium,* *Collinsella*, *Prevotella* and *Bacteroides* genera. The alpha indexes showed a decrease in microbial diversity after 6 h of colonic fermentation. The coordinates analysis indicated any differences between irradiated and non-irradiated bar. The metabolic prediction demonstrated that MangoB and UVMangoB increase the microbiota carbohydrate metabolism pathway. This study suggests that IF of mango-based bar induced beneficial changes on microbial ecology and metabolic pathway that could be promissory to prevention or treatment of metabolic dysbiosis. However, in vivo interventions are necessary to confirm the interactions between microbiota modulating and intestinal beneficial effects.

## 1. Introduction

In the tropical regions around the world, different native and exotic fruits are produced with a variety of colors and pleasant flavors, but these also have unique nutritional and therapeutic properties [1]. Mexico is a country with unique tropical fruits, including ‘Ataulfo’ mango (*Mangifera indica* L.) from the Soconusco state of Chiapas. This variety is popular due to its consistency, plump and sweet drupe, low acidity and intense aroma [2]. 

In general, mangoes are a good source of water, simple and complex carbohydrates, vitamins A and C, as well as diverse phytochemical compounds as phytosterols, carotenes or phenolic compounds (PCs). Dietary fiber (DF) is the major complex carbohydrate and its constituents in mango are pectin, cellulose, hemicelluloses [3] and klason lignin [4], while the major phytochemical substances are diverse PCs such as gallic, hydroxicinnamic, p-hydroxibenozoic, chlorogenic, caffeic and coumaric acids [5]. DF and PCs constitute the major components of the indigestible fraction (IF) of a food matrix. These represent substances that are not hydrolyzed by human gastrointestinal enzymes [6]. The IF can reach the large intestine and act as a substrate for colonic microbiota, releasing several metabolites such as short chain fatty acids (SCFA) and PCs that could be bioconverted and could induce microbial ecology changes [7,8,9]. During the fermentation process, gut bacteria can produce a wide range of compounds that have both positive and negative effects on gut physiology as well as other systemic influences [10]. It has been demonstrated that diet components such as DF, PCs or metabolic products can modulate the intestinal microbiota composition by stimulating beneficial specific bacteria and inhibiting pathogens [11]. Recently, changes in gut microbiota of mango peels in a dynamic large intestine fermentation system (TIM-2 model) have been reported. The main genera found were *Bifidobacterium*, *Lactobacillus*, *Dorea* and *Lactococcus*. Abundance was dependent on fermentation time, whereby *Bifidobacterium* were the most abundant at 24 h of fermentation [8].

According to Blancas-Benitez et al. [12], the paste and peel of ‘Ataulfo’ mango contain about 14.97 and 41.34 g/100 g dry weight (DW) of DF respectively. Additionally, paste and peel contains 10.7 and 12.7 g gallic acid equivalent (GAE) /100 g DW of PCs, associated with the total DF respectively. In view of this content, Hernández-Maldonado et al. [13] have recently developed a mango-based bar formulated with the “Ataulfo” peel and pulp, where they reported a content of 31.85 g/100 g DW of total DF and 14.35 g GAE/100 g DW of PCs in the bar. The in vitro gastrointestinal digestion showed 38.7 g/100 g DW of total IF, as well as 41.8 g GAE/100 g DW and 60.9 g GAE/100 g DW of PCs in the soluble and insoluble IF fractions, respectively. Furthermore, the colonic fermentation showed changes in production of acetate, propionate, butyrate and diverse phenolic acids [13]. However, changes in the structure and functionality of bacterial community during mango-based bars colonic fermentation are unknown. 

In the last decade, several investigations have focused on investigating the impact of pre-digested isolated fractions of foods or beverages on the intestinal microbiota during colonic fermentation. Studies include use of black tea and red wine [14], kiwifruit [15], pomegranate [16], apple varieties [11] and sugarcane [17]. These have reported a specific alteration on the structure and dynamics of the intestinal microbiota community, which are dependent on the isolated fraction source. These modifications could have repercussions on intestinal physiology.

The intestinal microbiota plays an important role in the physiological well-being of people, which is related directly to stimulation by bacterial cell components and the effects of bacterial metabolites [18]. Various approaches have focused on studying the intestinal microbiota either by quantitative techniques of microorganism culture [9] or by quantifying 16s rRNA fragment as a marker-gene. Marker-gene techniques include denaturing gradient gel electrophoresis (DGGE) [19], terminal restriction fragment length polymorphism (T-RFLP) [20], quantitative PCR (qPCR) [21], fluorescence in situ hybridization (FISH) [22] or plasmid-clone capillary Sanger sequencing [23]. However, for complex and diverse ecosystems such as intestinal microbiota, the previously mentioned methods have provided incomplete profiles of microbial community structure. High-throughput molecular technologies have recently elucidated microbial community structure at a much higher resolution [24]. Next generation sequencing (NGS) technologies have allowed the identification of multiple culture and non-culture microorganisms. Among these methods is Illumina MiSeq, a high-throughput molecular technology with greater use for colonic microbiota identification, based on sequencing data from high resolution and depth. 

Knowledge about the modification of microbiota due to the consumption of DF and PCs in the diet is limited. The aim of this work was to analyze the effect of the indigestible fraction from a mango-based bar on the colonic microbiota and metabolic output through in vitro colonic fermentation. Illumina Miseq 2 × 300 technique was used for this purpose. In addition, the effect of UV-C irradiation of mango-based bar on colonic microbiota was also investigated.

## 2. Materials and Methods 

### 2.1. Mango-Based Bar Sample 

The snack of mango peel and pulp was prepared according to Hernández-Maldonado et al. [13]. Briefly, “Ataulfo” mango was purchased at a local market in Tepic, Nayarit, Mexico. The whole fruits were washed with tap water, disinfected, and cut into 3 mm slices without removing the peels (Tor-Rey, R-300, Monterrey, México). Mango slices were separated into two batches. One batch was irradiated with UV-C (UVMangoB) in a chamber (38 cm × 60 cm × 78 cm; thickness/height/width) equipped with two fluorescent lamps and a UV light (60 cm × 5 cm., 15 W) twin-lamp (254 nm) (Germicidal, G15T8, Hiratsuka, Kanagawa, Japan) and the second batch was not irradiated (MangoB). The slices were placed 8 cm above and below the radiation rack, and each side was exposed for 10 min. The non-irradiated and irradiated mango slices were dehydrated in a forced air convection oven (70 °C, 22 h; Scorpion Scientific, A-52055, Mexico). The dried slices were then ground in a food processor (Nutribullet, NBR-0804B, CA, USA), shaped with a binding agent (Nutriose FB, Tecnovam, CDMX, Mexico), and dried again (60 °C, 3 h, Scorpion Scientific, A-52055, Mexico). Finally, slices were packed in trilaminate bags (Lamitec, UC1L-Z, Puebla, Mexico), frozen at −20 °C and freeze-dried (Labconco, Freezone 6–7752020, Kansas City, Missouri, United States), and subsequently ground (NutriBullet, NBR-0804B, CA, USA), sieved (0.5 microns), and stored in sealed bags at −20 °C until analysis. The details of the nutritional composition of the mango bar can be found in Hernández-Maldonado et al. [12].

### 2.2. Isolation of Indigestible Fraction (IF) in Mango Snack

The isolation of the IF was carried out as described by Tabernero, Venema, Maathuis and Saura-Calixto [25], following a gastrointestinal digestion in the ground freeze-dried mango-based bar. Briefly, the samples were mixed into pepsin solution (300 mg of pepsin/mL in 0.05 M HCl-0.03 M KCl buffer, pH 1.5, P-700, Sigma-Aldrich, St Louis MO, USA) and incubated with agitation at 40 °C for 1 h. Afterwards, a phosphate buffer was added and pH adjusted to 7.5. After pH adjustment, 1 mL of pancreatin solution (5 mg of pancreatin/mL in 0.1 M phosphate buffer, pH 7.5, P-1750, Sigma-Aldrich, St Louis MO, USA) was added and incubated and shaken at 37 °C for 6 h. Later, an α-amylase solution (120 mg/mL in 0.1 M tris maleate buffer, pH 6.9, A-3176, Sigma-Aldrich, St Louis MO, USA) was added and incubated at 37 °C for 16 h. The insoluble IF was removed by centrifugation (3000× *g*, 25 °C, 15 min) and washed twice with distilled water. The supernatant was mixed with sodium acetate buffer (pH 4.75) and 100 μL amyloglucosidase (A-9913, Sigma-Aldrich, St Louis MO, USA), and then incubated at 60 °C for 45 min. The solution was transferred into 12.4 kDa dialysis tubes 25 cm in length (D9652, Sigma-Aldrich, St Louis MO, USA) against water (7 L/h) for 48 h at 25 °C. The soluble IF was isolated by rotary evaporator at 60 °C until a final concentrate volume of 10 mL was obtained. The lyophilized insoluble IF + soluble IF mixture was denominated as total IF and used for colonic fermentation.

### 2.3. Fecal Microbiota Standardization 

The fecal samples (10 g) were donated by three healthy volunteers (ages 20–29 years old) who declared a normal diet, with body mass index (BMI) between 18 and 25. Volunteers declared that they did not follow any dietary restrictions, had no gastrointestinal diseases and had not taken antibiotics for last three months. A mandatory restriction was that subjects were not allowed to eat mango fruit or their derivatives one week prior to testing. Feces were anaerobically homogenized with 90 mL of 0.1 M phosphate buffer (pH 7.0) in an Ultra-Turrax T18 (Ika, Staufen im Breisgau, Germany). The mixed fecal sample was frozen in liquid nitrogen and stored at −80 °C until fermentation use. Donors were informed of the objectives of the study and they authorized the use of their fecal matter for the experiments.

### 2.4. In Vitro Colonic Fermentation

Total IF (500 mg) from UVMangoB and MangoB bars was fermented at 37 °C according to Zamora-Gasga et al. [7]. In addition, raffinose (R-0514, Sigma-Aldrich, St Louis MO, USA) was used as a positive fermentation reference (PositiveC) and fecal inoculum without substrate served as the negative control (NegativeC). Fermentations were done in triplicate in tubes containing 1 mL of fecal inoculum and 9 mL of culture medium (2 g/L peptone water, 2 g/L yeast extract, 0.1 g/L NaCl, 0.04 g/L K_2_HPO_4_, 0.04 g/L KH_2_PO_4_, 0.01 g/ L MgSO_4_-7H_2_O, 0.01 g/L CaCl_2_-2H_2_O, 0.01 g/L NaHCO_3_, 0.5 g/L cysteine HCl, 0.5 g/L bile salts and 2 mL/L between 80 and 0.2 g hematin (diluted in 5 mL of NaOH)) fermentation in order to maintain anaerobic conditions. The samples were monitored at 0, 6, 12, 24 and 48 h during fermentation. A total of 60 samples were stored at −80 °C for subsequent determination of metagenomic analyses. 

### 2.5. DNA Extraction and Library Construction

Of the colonic fermentation sample 200 µL was centrifuged at 12,000× *g* for 10 min. The pellet was then washed in 1 mL of phosphate buffer solution (PBS; 150 mM NaCl, 10 mM Na_2_HPO_4_, 10 mM NaH_2_PO_4_, pH 7.4). After washing, the sample was centrifuged at 12,000× *g* for 10 min and resuspended in 200 µL of filtered tris-ethylenediaminetetraacetic acid–SDS (TES) buffer pH 8 [10 mM Tris-HCl, 10 mM EDTA, 0.5% SDS]. The DNA was extracted according to the DNEasy™ Blood and Tissue Handbook (Cat. 69506, QIAGEN™, Hilden, Germany) protocol. Briefly, the previously washed sample was mixed with 250 µL of enzymatic lysis buffer (20 mM Tris-Cl pH 8.0, 2 mM EDTA, 1.2% Triton™ X-100, 20 mg/mL Lysozyme), vortexed and incubated at 37 °C for 30 min. Then, 180 µL of ATL™ buffer were added and gently mixed by inversion. Afterwards, 25 µL of proteinase K were added, mixed thoroughly and incubated at 56 °C for 40 min. Subsequently, 200 µL of AL buffer were added and mixed for 15 s by vortexing. The sample was centrifuged at 8000× *g* for 1 min and the supernatant was mixed with 200 µL of ethanol 96% in a clean microcentrifuge tube. The mixture was transferred into DNeasy Mini spin column, placed in a 2 mL collection tube and centrifuged at 6000× *g* for 1 min. The flow-through was then discarded, after which 500 µL of AW1™ buffer were added to DNeasy Mini spin column and centrifuged at 6000× *g* for 1 min. Upon discarding the flow-through again, 500 µL of AW2™ buffer were added to DNeasy Mini spin column and centrifuged at 15,000× *g* for 3 min, thus drying the DNeasy membrane. The DNeasy Mini spin column was placed in a new 1.5 mL microcentrifuge tube, to which 50 µL of AE™ buffer were directly added in the DNeasy membrane. Tubes were incubated at room temperature for 1 min and centrifuged at 6000× *g* for 1 min to elution. DNA purity and concentration were analyzed with NanoDrop™ One (ThermoFisher Scientific, Waltham, MA, USA). Then, 2 µL of isolated DNA were mixed with SYBR Safe DNA gel stain (Invitrogen Life Technologies Carlsbad, CA, USA), and run in 1% (w/v) agarose gel electrophoresis and visualized by use of UV Transilluminator 2000 (BIO-RAD Laboratories Inc., CA, USA). The 1-Kb DNA ladder (Invitrogen Life Technologies Carlsbad, CA, USA) was used as a molecular size marker. The extracted DNA was stored at −20 °C until required for PCR amplification and sequencing. The variable V4-V5 regions of the 16s rRNA gene of the total DNA extracted from monitoring samples were sequenced in CGEB-Integrated Microbiome Resource (IMR) at Dalhousie University (Halifax, NS, Canada). The 515F (5’-GTGYCAGCMGCCGCGGTAA-3’) and 926R (5’-CCGYCAATTYMTTTRAGTTT-3’) were primers used [26]. Barcoded DNA libraries were sequenced through Illumina (San Diego, CA, USA) MiSeq 2 × 300 bp platform. 

### 2.6. Bioinformatics Analysis 

Demultiplexed raw sequence data were unzipped and analyzed for quality with FastQC of raw paired-ends reads [27]. The Usearch [28] and Vsearch [29] tools were used for progressive genomic analysis through the marker-gene workflow described by Lee, M. [30]. Forward and reverse reads were merged using fastq_mergepairs to create a total consensus sequence. Subsequently, the primers were trimmed at 19 bp to the left and 20 bp to the right of each sequence according to the respective bp length of the primers used. The quality filtering was done using fastq_filter based on ± 10% of the expected length of sequences, while fastq_maxlen 415 and fastq_minlen 330 was applied. All identical sequences were collapsed to one representative using the derep_fulllength function. Then, error amplicons and chimeras were removed with unoise3 algorithm. A 0.001% minimum abundance threshold was set to create the Amplicon Sequence Variants (ASVs) table. A count table was created using the usearch_global with 0.99 of threshold similarity. The assigning of taxonomy to our ASVs sequences was performed with sintax on a non-Bayesian taxonomy classifier and using the SILVA high quality ribosomal RNA database (silva_16s_v123.fa). Furthermore, the computational approach of phylogenetic was used for investigation of communities by reconstruction of unobserved states (PICRUSt) from Langille et al. [31], based on Kyoto encyclopedia of genes and genomes (KEGG) from Kanehisa et al. [32]. This was employed in order to predict the functional composition of the metagenome. 

### 2.7. Statistical Analysis 

Experiments were performed by triplicate and mean values with standard deviations were calculated. DNA quantity results were subjected to a one-way analysis of variance (ANOVA) and significant differences were calculated using least significant difference (LSD) Fisher’s in Statgraphics Centurion XVI (Statgraphics Technologies, Inc., VA, USA). Alpha-diversity based on full amplicon sequence variants (ASVs) table was calculated using R studio (version 1.2.1335, Inc., Boston, MA, U.S.A) vegan package [33]. This helped to determine the microbial rarefaction curves, Chao1, ACE, Simpson and Shannon indexes. Later, downstream analysis was performed, based on the mean values of the ASVs data triplicates. The bar plot visualization was constructed with a relative abundance of the genus level > 0.05% of ASVs count table and rest summarized in others. The hierarchical clustering analysis was performed based on the Euclidean distance matrix of genus > 0.05% ASVs table and visualized in dendrograms using the dendextend package [34]. The principle coordinate analysis (PCoA) was calculated by the multidimensional scaling method (MDS) and Euclidean distance similarity using phyloseq R/Bioconductor library, which was later visualized using ggplot2 library [35]. Permutational multivariate analysis of variance (PERMANOVA) was conducted using XLSTAT 2019.4.2, based on the Euclidean distance (*p* > 0.05) with 9999 permutations (Addinsoft Inc., New York, NY, USA). 

## 3. Results and Discussion

### 3.1. Extracted DNA and Amplicon Sequence Variants (ASVs) Results 

Total DNA extraction was successful with the QIAGEN™ protocol for all colonic fermentation samples. The quantity and quality of DNA obtained among treatments and times is shown in Table 1. In general, the DNA concentrations in colonic fermentation samples of UVMangoB, MangoB and PositiveC samples (*n* = 3) were higher, compared with NegativeC. The DNA-based microbial biomass estimation has been reported to be a highly effective and suitable alternative to basic approaches for microbial biomass determination [36]. Therefore, the increase in DNA concentration from 3-8 to 173–249 ng/µL between samples suggests that changes in fecal microbial composition take place with passing of colonic fermentation times. This was compared with the unchanged NegativeC where the maximum DNA concentration was 6 ng/µL after 12 h. These levels had been initially 3 ng/µL.

After sequencing by Illumina Miseq 2 × 300 platform, a total of 1,734,185 Illumina paired-ends reads were obtained, which corresponded to the V4–V5 hypervariable region of the 16 s rRNA gene. The alignment of forward and reverse pairs resulted in 1,060,472 of successful consensus data that overlapped along the reads. Overhangs at the ends of the reads, which did not align for both were discarded. After filtering, 1,039,916 sequences were kept, and 20,556 sequences discarded due to poor quality (spurious) or length of expected sequences. Thus, all sequence dereplication collapsed down to 206,011 identical sequences and the most abundant sequences were in 129,480 copies. The filter by a minimum abundance threshold of 0.001% of total sequences and removal of suspected chimeras resulted in 557 ASVs as a unique and true biological sequence from 60 libraries. 

The sequences obtained in ASVs files were taxonomically classified using the Silva reference database. Afterwards, chloroplast assigned reads were removed manually. Table 1 shows the number of ASVs located in each fermentation time by sample as well as the number of reads located into ASVs. Despite initial DNA concentration between treatments differing by up to two orders of magnitude (from 3 to 249 ng/µL of sample), the number of ASVs and sequences per treatment was very consistent at 216 ± 57 ASVs and 12,785 ± 8591 sequences (average ± standard deviation, *n* = 60), respectively.

### 3.2. Bacterial Community Diversity in Colonic Intestinal Fermentation 

**Figure 1:**
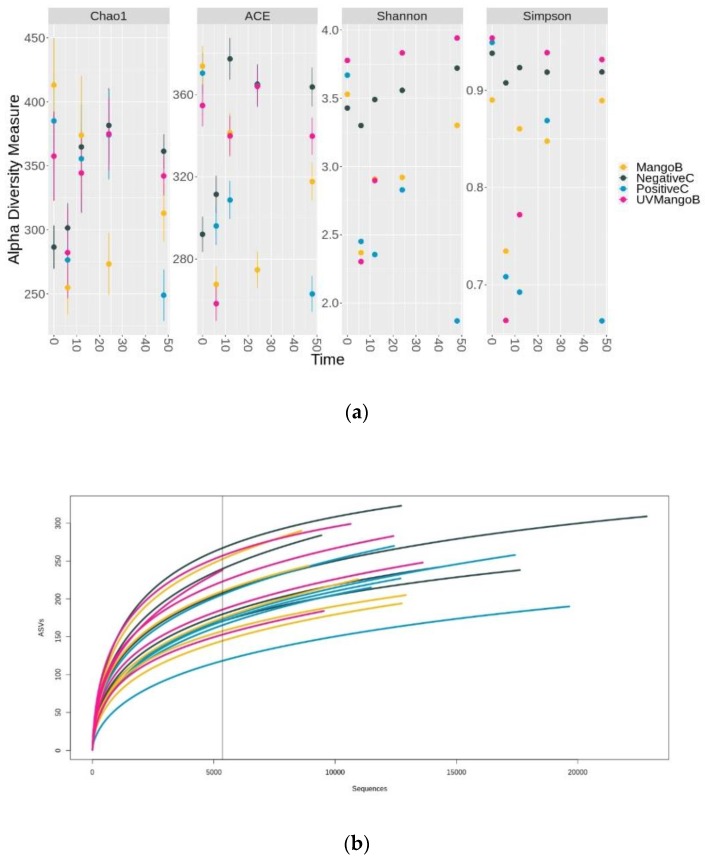
Point chart and rarefaction curves of amplicon sequences variants (ASVs) from in vitro colonic fermentation samples of negative control (NegativeC), positive control (PositiveC), UV-C irradiated mango bar (UVMangoB) and non-irradiated mango bar (MangoB) treatments. (**a**) Alpha-diversity estimators by richness (Chao1 and ACE) and diversity (Shannon and Simpson) indexes of observed species into ASVs at 0, 6, 12, 24 and 48 h of colonic fermentation. (**b**) Rarefaction curves based on the ASVs from colonic fermentation of NegativeC (grey dark), PositiveC (blue), MangoB (yellow) and UVMangoB (pink). The x-axis indicates the number of valid sequences and the y-axis shows the observed species in ASVs. Each curve represents a different monitoring sample and the vertical straight line indicates the minimum number of sequences found in a sample.

Based on 97% of the sequence identity threshold, the bacterial 16s rRNA marker gene reads were taxonomically assigned to obtain the microbial community composition. A total of 557 different ASVs were found among treatments, with 455 detected in NegativeC, 425 in PositiveC, 476 in MangoB and 476 in UVMangoB. The alpha richness indexes (Chao1, ACE, Shannon and Simpson), based on the number of observed ASVs, are shown in Figure 1a. These estimators showed a fluctuating diversity of fecal microbiota composition during the colonic fermentation process. The NegativeC treatment (only inoculum) presented the most stable microbial structure during the 48-h study. It showed the minimum Chao1 value at time 0 and maximum at the final sampling time. Their Shannon index remained unchanged. However, the Chao1 and Shannon diversity indexes dropped significantly after 6 h for PositiveC, MangoB and UVMangoB treatments. The richness in diversity of these samples was gradually recovered after 12 h of fermentation, whereby negative control levels were reached at 48 h, except for PositiveC, where diversity drops again after 24 h. Similar reduction of Chao1 and Shannon indexes during colonic fermentation of apple varieties were reported by Koutsos et al. [11]. Grant et al. [37] have reported the increase of microbial diversity indexes in the large intestine of porcine cattle fed with a mango-diet, whereby a reduction of diversity indexes was observed for those fed with wheat pectin-diet and starch-diet. These results indicated a selective nature of microorganisms, which is influenced by substrates, metabolic machinery capacity and the microbial host of fecal composition. 

The rarefaction curves represent the diversity as a function of sequencing depth. Figure 1b shows that species increased with the advance of the sequencing until approaching asymptotic behavior, indicating that microbiota structure and diversity were represented and covered very well as reported by Serra et al. [38]. Hence, further sequencing would not result in more ASVs identification by each sample.

### 3.3. Changes of Microbial Composition during Mango (Mangifera indica L.) IF Colonic Fermentation

The mean values of the ASVs data triplicates presented 2 domains, 9 phyla, 17 classes, 27 order, 46 families and 101 genera. These results showed that 96% of microorganisms belonged to Firmicutes (55%), Proteobacteria (29%), Actinobacteria (11%) and Bacteroidetes (1%) phyla (Figure 1a). The remaining 4% included other phyla or unclassified bacteria with low abundance (<0.05%) during the colonic fermentation process. 

A different phyla composition has been reported by Duda-Chodak et al. [39] who indicated that 98% of all species in the human gut belong to Firmicutes (64%), Bacteroidetes (23%), Proteobacteria (8%) and Actinobacteria (3%), as well as other minor taxonomic divisions. However, it was found that composition is strongly dependent on the intestinal location and the ethnicity of the people.

In this study, 0.03% belonged to the Archaea domain, with 0.02% pertaining to Thaumarchaeota and 0.01% pertaining to Euryarchaeota phyla, classified within *Nitrososphaera* and *Methanobrevibacter* genus, respectively. Thus, *Nitrososphaera* remained unchanged for all treatment times, whereas *Methanobrevibacter* increased in the last sampling times of NegativeC but was totally absent in the PositiveC, MangoB and UVMangoB treatments. *Nitrososphaera* is an ammonia-oxidizing and degraded urea member associated with the ingestion of proteins and amino acids from the diet [40]. As for *Methanobrevibacter*, it is the main human methanogen that is almost always found in the digestive tract of adults, with colonization taking place just after birth. *Methanobrevibacter* is also the main CH_4_/H_2_O producer, which is carried out by transforming H_2_ and CO_2_ [41]. 

**Figure 2:**
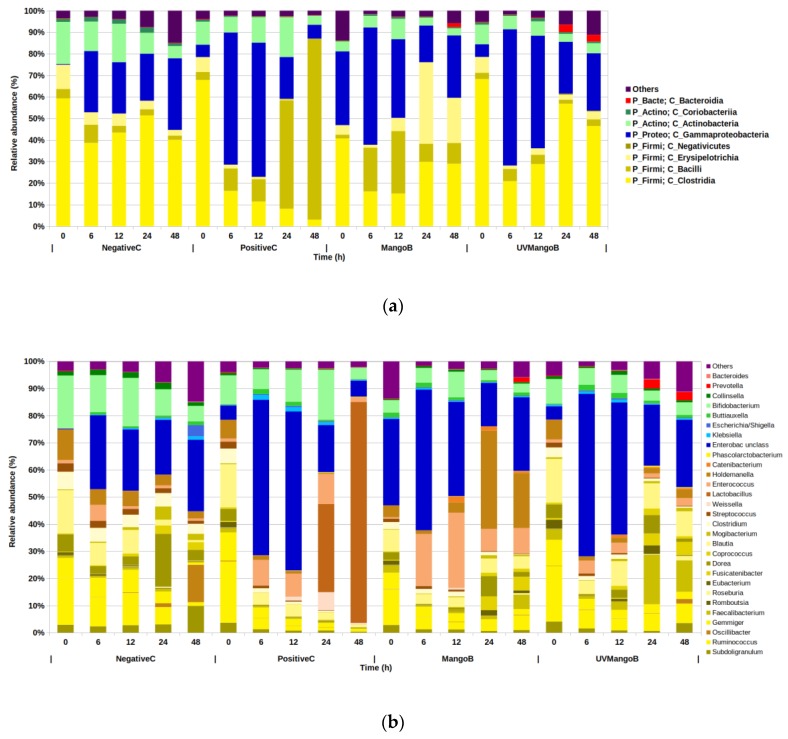
Relative abundance of bacterial composition determined by 16 s rRNA (V4-V5 region) Illumina sequencing at 0, 6, 12, 24 and 48 h of colonic fermentation in negative control (NegativeC), positive control (PositiveC), non UV-C irradiated mango bar (MangoB) and UV-C irradiated mango bar (UVMangoB) treatments. Percentage mean (*n* = 3). (**a**) The most abundant phylum and class level are depicted by Firmicutes (yellow), Proteobacteria (blue), Actinobacteria (green), Bacteroidetes (red) and Others (purple). (**b**) At the genus level, differing shades of phylum’s colors were applied. Genera > 0.05% of the total ASVs composition were considered for the graph.

The relative abundance was mainly represented by 4 phyla, 8 classes and 29 genera of bacteria domains found during colonic fermentation (Figure 2b). In NegativeC, we observed that Bacteroidetes remained unchanged. However, after 48 hours, alterations in Firmicutes (from 75% to 45%), Proteobacteria (from 0.3% to 33%) and Actinobacteria (from 21% to 7%) were observed. This indicated that changes in fecal bacterial composition would exist over time, regardless of any substrate incorporated. Similar behavior of the fecal inoculum control has been observed in similar colonic fermentation systems [15]. In the case of raffinose treatment as a colonic fermentation reference, we observed Bacteroidetes (from 0.2% to 0.1%) relatively unchanged along the 48-hour study. However, Actinobacteria and Proteobacteria increased and Firmicutes decreased during the first 12 h (from 11% to 18%, from 6% to 62% and from 79% to 23%, respectively). After 24 h the behavior was inverse. Adamberg et al. [42] reported similar behavior in microbial abundance in raffinose fermentation by using pooled fecal samples from overweight and normal-weight children.

The colonic fermentation of indigestible fraction of mango-based bar (MangoB) showed that the Bacteroidetes composition was not modified until the end of the fermentation, where a growth was observed (48 h). However, Proteobacteria, Actinobacteria and Firmicutes revealed a substantial increase at earlier times (6, 12 and 24 h respectively), whereas their abundance was reduced for the rest of fermentation times. Recently, the colonic fermentation of pre-digested mango peels showed that Bacteroidetes phylum was not promoted, whereas the growth was favorable for Firmicutes and Actinobacteria [8]. In this work, the fermentation of the indigestible fraction isolated from UV-C irradiated mango-based bar (UVMangoB) increased the abundance of Proteobacteria and Actinobacteria during the first 12 h, with an increase of Firmicutes and Bacteroidetes from 6 h until 24 h. The four phyla were reduced in the final hours of the process. There has been an increased interest in using UV-C light for bactericidal and antifungal treatments of foods [43], and it is considered a feasible treatment for polyphenol oxidase (PPO), peroxidase (POD) and phenylalanine ammonia lyase (PAL) activities [44]. However, the effect of UV-C light on substrates for colonic microbiota remains unclear. 

The higher phylum assigned was Firmicutes, which presented 4 classes and 21 genera (out of 28 total) higher than 0.05% relative abundance. Clostridia were the most abundant class, followed by Bacilli, Erysipelotrichia and Negativicutes (Figure 2b). Pertaining to the Clostridia class, the genera *Ruminococcus*, *Oscillibacter*, *Gemmiger*, *Coprococcus* and *Mogibacterium* were considerably reduced during the 48-hour study in the PositiveC, MangoB and UVMangoB, as compared to the NegativeC. With *Subdoligranulum*, *Rombutsia* and *Clostridium* belonging to the same Clostridia class, these genera only showed a reduction in abundance for MangoB and UVMangoB treatments. This decrease could be related to the inhibition effect of polyphenols and their metabolites on some pathogenic and non-pathogenic gut microbial bacteria, as reported by Duda-Chodak et al. [39]. These Gram-positive microorganisms have shown to be more susceptible to polyphenols than Gram-negative bacteria, possibly due to differences in their wall composition, which makes them more vulnerable to having altered permeability in the microbial membrane [45]. In contrast *Dorea*, *Blautia*, *Faecalibacterium*, *Roseburia*, *Eubacterium* and *Fusicatenibacter* showed an increase in MangoB and UVMangoB treatments. In other research, the colonic fermentation of mango peel showed an increased abundance of *Dorea* and *Blautia*, both involved in complex carbohydrates digestion [8]. *Dorea* could be the principal acetic acid producer during colonic fermentation reported by Hernández-Maldonado et al. [13]. Even, members of the Lachnospiraceae family have been reported as a responsible of *C*-glucoside mangiferin deglycosylation for the production of norathyriol [46,47]. *Dorea* is a microorganism belonging to Lachnospiraceae family and could be related with the mangiferin biotransformation reported by Hernández-Maldonado et al. [13]. On the other hand, *Faecalibacterium*, *Roseburia* and *Eubacterium* genera are involved in the fermentation of pectin oligosaccharides [48] and they are the main butyrate producers via Butyryl-CoA in the colon [49]. This could be very important because emerging studies have demonstrated that gut microbiota from individuals suffering from inflammatory bowel disease (IBD) have a decreased abundance of butyrate-producing bacteria (BPB), such as *Roseburia hominis*, *Faecalibacterium prausnitzii* and *Eubacterium rectale* [50].

As for the Bacilli class, the abundance of *Streptococcus* genus is reduced in all experimental treatments during the 48 h experiment, as compared to the NegativeC basal statement. On the other hand, *Weissella* and *Lactobacillus* increased significantly only in the PositiveC in the last 24 h. Different species of *Streptococcus* genus have been identified as fructose and raffinose-metabolizing in fecal samples [51]. However, in this work the unclassified specie of *Streptococcus* was not particularly plentiful. Similarly, *Lactobacillus* is a widely reported genus that comprises of several probiotic species that can metabolize raffinose carbohydrate and oligosaccharides [52]. *Weissella* genus is reported as possessing phosphotransferase systems of β-glucosidase and α-galactosidase genes for metabolizing D-cellobiose and D-raffinose [53], but their metabolic capacity was unobserved. In the case of the *Enterococcus* genus belonging to the Bacilli class, we observed a lower presence in NegativeC but higher prevalence in the MangoB treatment. Although species such as *Enterococcus faecalis* have been reported as responsible of health problems in the human gut [54], other species such as *Enterococcus faecium* have been reported as beneficial probiotic microorganisms with the capacity of starting fermented cultures, high acidity and bile salt tolerance that could contribute to reducing serum cholesterol levels [55]. Recently, Wei et al. [56] reported that some species of the *Enterococcus* and *Eubacterium* genera, have been the guiding forces in the biotransformation of C-C glycosidic bonds, which are present in flavone, xanthone and phenolic acid biomolecules. They also suggested that cleavage is not a simple one-step hydrolysis reaction, but a type of complex enzymatic biotransformation. The biotransformation of xanthone C-glycosides (mangiferin and norathyriol), flavone C-glycoside (quercetin, catechin and gallocatechin), hydroxycinnamic and hydroxybenzoic acids from the indigestible fractions of the mango-based bar has been demonstrated previously by Hernández-Maldonado et al. [13]. 

At the species level, *Catenibacterium mitsuokai* and *Holdemanella biformis* were the two more abundant species belonging to the Erysipelotrichia class. The members of this taxa, generally considered as a Gram positive and anaerobic microorganisms, remained unchanged in the negative control, but declined significantly for PositiveC and increased in mango IF (both MangoB and UVMangoB) fermentation. After 12 h, *Catenibacterium mitsuokai* accounted for up to 2% of total abundance in MangoB and UVMangoB treatments, suggesting that it could have important metabolic characteristics. Unclassified microorganisms of this genus have been reported as predominant during the colonic fermentation of agave fructans and oat bran β-glucan [57]. They have also been reported during carrot DF and bound polyphenols for carrot DF digestion reported by Liu et al. [58]. In the case of *Holdemanella biformis*, a considerable increase was observed in the last 24 h in MangoB fermentation but not in UVMangoB treatment. This could indicate an important metabolism for DF or polyphenolic metabolites and possible long-term effect of UV-irradiation. This genus has recently been described as positively correlated with butyric and acetic acid, and negatively correlated with ammonium ions during obesity-related microbiota research [59].

No Negativicutes class members were detected in the NegativeC (regardless of time) and at early times of all other treatments. However, *Phascolarctobacterium succinatutens*, the main propionate-producing specie, was detected after 12 h in PositiveC, MangoB and UVMangoB treatments, rising to 0.5% of total abundance. This suggests that pyruvate and phosphoenolpyruvate coming from the catabolism of dietary fiber are converted to succinate by Bacteroidetes members, being then converted to propionate *P. succinatutens*, via the succinyl-propionyl-CoA pathway [49,60].

The second dominant phylum was Proteobacteria, from which only the Gammaproteobacteria class and three genera were found. In general, this phylum houses more than 450 species of Gram negative microorganisms and a wide variety of pathogens [61]. Similar to our results, the increased proportion of Proteobacteria has been reported in colonic fermentation of kiwifruit [15] and apple varieties [11]. This has also been observed for unclassified Enterobacteriaceae families during black tea and red wine colonic fermentation [14]. Here, we observed a minor abundance of *Escherichia*/*Shigella*, which are usually a genus found in fecal samples. Although the *Klebsiella* genus is generally considered to be a commensal opportunistic pathogen, the ability to metabolize epigallocatechin gallate and flaxseed lignans [14] make it beneficial for metabolic activity in MangoB and UVMangoB, where the relative abundance was as high as 0.5% and 0.8%, respectively. Interestingly, the increased presence of the *Buttiauxella* genus (around 1.5%) during MangoB and UVMangoB treatments could indicate a possible benefit due to its potential for phytase production. The degradation of phytate by *Buttiauxella* phytase and improved of amino acid digestibility have been reported by Dersjant-Li et al. [62]. This suggests that the presence of this microorganism could reduce the antinutritional phytate presence in mango peels reported by Romelle et al. [63].

*Bifidobacterium* and *Collinsella* were the most abundant genus belonging to the Actinobacteria and Coriobacteria class, respectively. They also represent the Actinobacteria phylum. *Bifidobacterium* is a Gram positive and anaerobic genus of microorganism that mainly in the gastrointestinal tract of mammals, birds and insects [64]. In this work, we observed a relative abundance of 5%-10% of *Bifidobacterium* during MangoB and UVMangoB treatments. The increased abundance of this genus has been reported for mango peels fermented in a TIM-2 dynamic system [8] and is considered a beneficial member of the gut microbiota by inhibiting the growth of pathogens, and because of its synthesis of certain vitamins and reducing of serum cholesterol [11]. That is also the case for *Collinsella*, a genus including more than nine species, which can modify host bile acids, plasma cholesterol levels and produce butyric acid. The alleviation of inflammatory bowel disease, type 2 diabetes, obesity and rise in energy sources for epithelial cells have been related to good butyrate production in colon [65].

*Bacteroides* and *Prevotella* were the main genera found belonging to the Bacteroidia class and Bacteroidetes phylum. Both genera have been reported with reciprocal patterns of abundance. *Bacteroides* correlated well with consumption of choline, fats and amino acids, while *Prevotella* correlated with the composition of carbohydrates [40]. However, a lower abundance of these genera (2% and 0.2%, respectively) was observed in this work. Notwithstanding, *Bacteroides* species have been reported as important members that are associated with lignan-polyphenol metabolism [66], and have also been described as possessing C-glycosyl bond-cleaving capabilities [56]. A similar low abundance of this genus during colonic fermentation of mango peels was observed by Sáyago-Ayerdi et al. [8]. 

The static system used for the fermentation of mango bars, shows some limitations, similar as any in vitro model, however many of the genera found in this study were similar to those published in a TIM-2 dynamic system. However, it is important to stress that the study of the whole diet has to be part of further research because it is the most common way to study consumed foods.

### 3.4. Sample Clustering and Principal Coordinate Analysis (PCoA) Analysis

**Figure 3:**
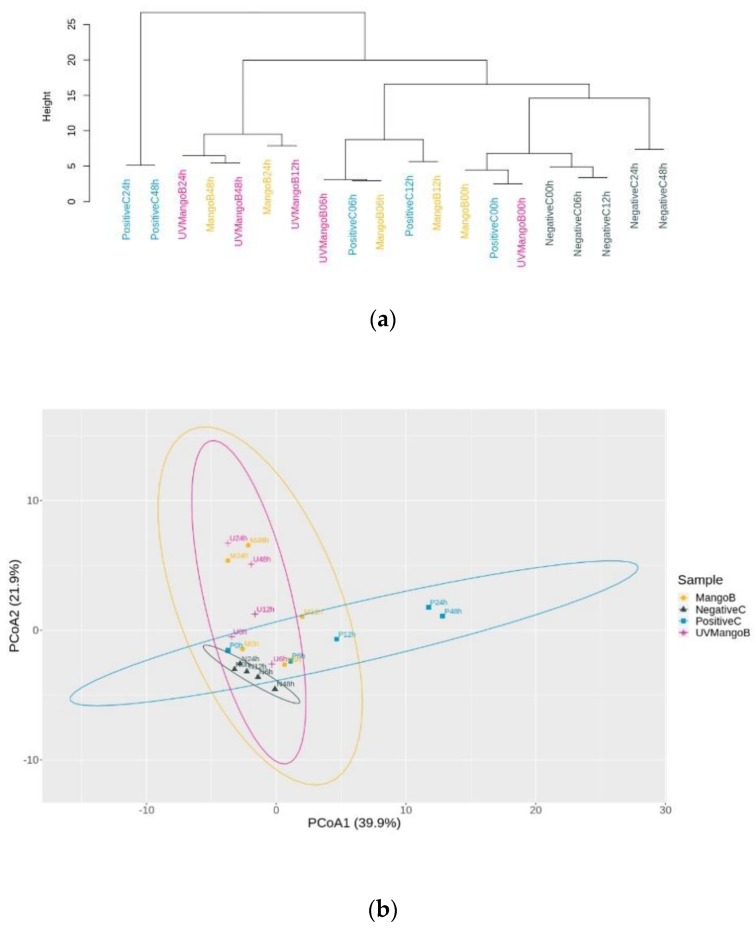
Clustering and principal coordinate analysis (PCoA) of observed species into amplicon sequences variants (ASVs) from in vitro colonic fermentation samples at 0, 6, 12, 24 and 48 h of negative control (NegativeC), positive control (PositiveC), UV-C irradiated mango bar (UVMangoB) and non-irradiated mango bar (MangoB) treatments. (**a**) Hierarchical clustering dendrogram based on the Euclidean distance of genera abundance higher that 0.05%. (**b**). First two eigenvalues (PCoA1 and PCoA2) based on the Euclidean distance matrix of ASVs found during 48 h of colonic fermentation.

The dendrogram based on the Euclidean distance clustering analysis (Figure 3a) showed the relationship of evolutionary fermentation between colonic fermentation samples. All initial fermentation times were clustered in the same root, which indicated a similar distance of microbial abundance of ASVs at the beginning of fermentation. Later, MangoB, UVMangoB and PositiveC treatment increased in matrix distance abundance, interposing the clustering for some microbial members. Although the inoculum samples showed an increase in ASVs in the final hours, clustering on the opposing side demonstrated differences in the microbial composition.

The PCoA (Figure 3b) revealed that the two eigenvalues (PCoA1 with 39.9% and PCoA2 with 21.9%) reflected the variance represented by the contributory factors for the microbial composition of four treatments and five times. This analysis separated the PositiveC treatment towards PCoA1, while PCoA2 put together colonic fermentation samples of MangoB and UVMangoB, according to a similar abundance of microbial composition. Most samples from PositiveC were separated from NegativeC and MangoB in at least two of the coordinate combinations. The permutational multivariate analysis of variance (PERMANOVA) analysis confirmed that the major significant differences were obtained due to treatment samples (*F* = 2.769, *R*^2^ = 0.273, *p* = 0.001), as compared to the fermentation time (*F* = 3.718, *R*^2^ = 0.122, *p* = 0.002). A considerable significance was obtained for the sample–time interaction (*F* = 2.141, *R*^2^ = 0.211, *p* = 0.014).

### 3.5. Metabolic Prediction: Phylogenetic Investigation of Communities by Reconstruction of Unobserved State (PICRUSt) Analysis

**Figure 4:**
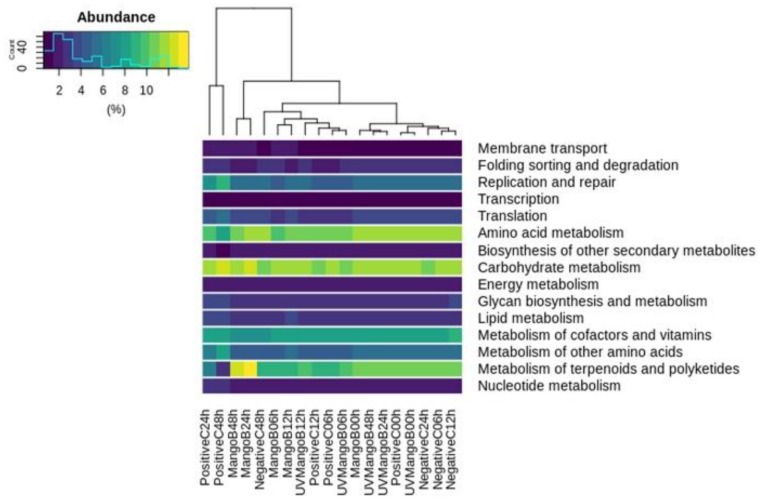
Heatmap of bacterial-predicted metabolic functionality based on phylogenetic investigation of communities by reconstruction of unobserved state (PICRUSt) analysis and Kyoto Encyclopedia of Genes and Genomes (KEGG). The rows indicate the KEGG level-2 pathway (>1% of abundance) and the columns show the hierarchical clusters of the negative control (NegativeC), positive control (PositiveC), UV-C irradiated mango bar (UVMangoB) and non-irradiated mango bar (MangoB) samples.

The PICRUSt resulted in a total of 124 Kyoto Encyclopedia of Genes and Genomes (KEGG) orthology (KO) level-3 pathways predicted from 16s rRNA sequences in terms of functional orthologs. Forty four KO level-3 pathways higher that 1% of abundance were collapsed in 15 KO level-2 pathways and plotted in Figure 4. These more abundant KO represent 71% of the total predicted process and belonged to metabolism, genetic information processing and environmental information processing, located in KO level-1.

We identified that carbohydrate metabolism, amino acid metabolism, metabolism of terpenoids and polyketides and metabolism of the cofactor and vitamins represented the most abundant pathways for KO level-2 between the samples. The majority of the NegativeC samples were grouped into the same cluster, suggesting lower changes in metabolic functions with time. Particularly, higher amino acid metabolism was observed during initial times of treatments and decreased according to fermentation progress for MangoB, UVMangoB and PositiveC. This was in contrast with the carbohydrate-metabolism pathway, which was lower at the initial times and increased later for MangoB and UVMangoB treatments. These results make it potentially beneficial for metabolic processes, as mentioned by Canfora et al. [67]. This last study reviewed that proteolytic fermentation generates products that are detrimental for health, and that saccharolytic pathways are more important for prevention and/or treatment of metabolic disease. On the other hand, although the abundance of metabolism of terpenoids and polyketides (KO level-2) was significantly greater after 24 h and 48 h of colonic fermentation for MangoB treatment, this increase could be related to the bioconversion of metabolites. The main level-3 pathways were related to biosynthesis of ansamycins and vancomycin-group antibiotics, which is normal for a niche where a large community of microorganism is constantly competing for growth. 

## 4. Conclusions

In this study, we demonstrated that despite fecal inoculum changes in NegativeC treatment, the IF of UVMangoB and MangoB mango-based bars exerted additional benefits for intestinal microbial composition. This work demonstrated that the IF of the ‘Ataulfo’ mango-based bar favored the growth of *Faecalibacterium*, *Roseburia*, *Eubacterium*, *Fusicatenibacter*, *Holdemanella*, *Catenibacterium*, *Phascolarctobacterium*, *Bifidobacterium*, *Collinsella*, *Prevotella* and *Bacteroides* genera microorganisms. All these microorganisms were strongly related to DF fermentation, production of metabolites and metabolism of PCs that induce positive effects for human health. MangoB and UVMangoB increased the *Bacteroidetes* phylum and decreased the Firmicutes/Bacteroidetes ratio, which is essential for restoration in dysbiosis case or in the maintenance of the microbial gut balance. Microbiota saccharolytic pathways were enhanced with IF from ‘Ataulfo’ mango and promised it to be beneficial for human gut health benefits. However, it is advised that findings should be contrasted with in vivo evaluations to asseverate these beneficial effects.

## Figures and Tables

**Table 1:** DNA concentration, Illumina sequencing reads and amplicon sequence variants (ASVs) during colonic fermentation of negative control (NegativeC), positive control (PositiveC), non-UV-C irradiated mango bar (MangoB) and UV-C irradiated mango bar (UVMangoB) treatments ^1^.

Treatment/ Time (h)	DNA (ng/µL of sample) ^2^	Ratio 260/280	Paired-End Reads	Number of ASVs	Number of Reads into ASVs
NegativeC					
0	3.33 ± 1.26 ^A, c^	1.36 ± 0.11	37,369 ± 44,721	186 ± 75	17,634 ± 22,852
6	4.12 ± 1.26 ^A, b^	1.50 ± 0.05	36,373 ± 12,886	192 ± 82	14,331 ± 3447
12	6.51 ± 3.65 ^A, c^	1.61 ± 0.32	46,199 ± 9255	256 ± 88	22,897 ± 27,685
24	5.69 ± 1.25 ^A, b^	1.46 ± 0.06	23,887 ± 9255	257 ± 39	9459 ± 3742
48	4.46 ± 1.43 ^A, b^	1.50 ± 0.01	32,985 ± 9351	290 ± 26	12,738 ± 3886
PositiveC					
0	8.10 ± 2.69 ^C, a^	1.79 ± 0.20	33,125 ± 18,765	239 ± 31	12,461 ± 7373
6	33.24 ± 20.33 ^B,C, a^	1.99 ± 0.22	26,111 ± 15,951	191 ± 32	11,510 ± 8355
12	22.56 ± 8.57 ^C, b,c^	1.99 ± 0.08	28,090 ± 10,072	210 ± 27	12,720 ± 4653
24	77.84 ± 41.85 ^B, a,b^	2.09 ± 0.04	40,410 ± 5110	241 ± 18	17,432 ± 3531
48	173.80 ± 37.93 ^A, a^	2.10 ± 0.07	37,155 ± 4676	175 ± 24	19,665 ± 2817
MangoB					
0	3.02 ± 0.46 ^C, c^	1.51 ± 0.42	21,467 ± 13,471	212 ± 84	9405 ± 5675
6	27.21 ± 3.04 ^C, a^	2.01 ± 0.05	25,853 ± 9343	184 ± 16	12,780 ± 4895
12	70.015 ± 37.54 ^B,C, a^	2.09 ± 0.11	23,745 ± 14,122	196 ± 26	10,960 ± 6581
24	155.96 ± 65.02 ^B, a^	2.18 ± 0.05	30,332 ± 7471	189 ± 23	12,939 ± 3135
48	249.02 ± 83.25 ^A, a^	2.19 ± 0.02	22,516 ± 7369	217 ± 34	8927 ± 3325
UVMangoB					
0	6.37 ± 3.55 ^C, b,c^	1.78 ± 0.11	13,274 ± 1153	129 ± 112	3538 ± 3080
6	24.30 ± 5.19 ^C, a^	7.80 ± 0.11	20,042 ± 11,747	179 ± 27	9578 ± 5704
12	53.27 ± 14.96 ^C, a,b^	1.84 ± 0.22	28,441 ± 5716	225 ± 17	13,631 ± 2283
24	134.00 ± 64.64 ^B, a^	2.15 ± 0.07	29,500 ± 3691	262 ± 8	12,447 ± 1313
48	196.82 ± 21.88 ^A, a^	2.18 ± 0.02	25,614 ± 2438	285 ± 8	10,648 ± 1041

^1^ Mean values ± standard deviation (*n* = 3). ^2^ Significant differences (*p* < 0.05) using ANOVA/Fisher’s least significant difference (LSD) test, are indicated in column by different capital letters (A, B, C) within monitoring times for each treatment. Lowercase letters (a, b, c) indicated in same column significant differences (*p* < 0.05) between similar monitoring times of treatments.
